# One potential biomarker for teratozoospermia identified by in-depth integrative analysis of multiple microarray data

**DOI:** 10.18632/aging.202781

**Published:** 2021-03-26

**Authors:** Baoquan Han, Lu Wang, Shuai Yu, Wei Ge, Yaqi Li, Hui Jiang, Wei Shen, Zhongyi Sun

**Affiliations:** 1 Urology Department, Peking University Shenzhen Hospital, Shenzhen Peking University and The Hong Kong University of Science and Technology Medical Center, Shenzhen 518036, China; 2 College of Life Sciences, Institute of Reproductive Sciences, Qingdao Agricultural University, Qingdao 266109, China; 3 Urology Department, Zaozhuang Hospital of Zaozhuang Mining Group, Zaozhuang 277100, China; 4 Department of Urology, Department of Andrology, Department of Human Sperm Bank, Peking University Third Hospital, Beijing 100191, China

**Keywords:** teratozoospermia, integrative analysis, GSEA, WGCNA, semen biomarker

## Abstract

Teratozoospermia is a common category of male infertility and with the increase in clinical patients and the increasing sophistication of assisted reproductive technology, there is an urgent need for an accurate semen diagnostic biomarker to accomplish rapid diagnosis of patients with teratozoospermia and accurately assess the success rate of assisted reproductive technologies. In this study, we performed gene differential expression analysis on two publicly available DNA microarray datasets (GSE6872 and GSE6967), followed by GSEA analysis to parse their enriched KEGG pathways, and WGCNA analysis to obtain the most highly correlated modules. Subsequent in-depth comparative analysis of the modules screened into the two datasets resulted in a gene set containing the identical expression trend, and then the differentially expressed genes in the set were screened using the corresponding criteria. Finally, three differentially expressed genes common to both datasets were selected. In addition, we validated the expression changes of this gene using another dataset (GSE6968) and *in vitro* experiments, and only screened one potential semen biomarker gene whose expression trend was identical to those in other datasets, which will also provide an important theoretical basis for the diagnosis and treatment of teratozoospermia.

## INTRODUCTION

Male infertility is one of the most commonly diagnosed conditions in reproductive health, which is multifactorial and accounts for about half of all infertility cases [1]. Among these male infertility diseases, teratozoospermia is a common category of male infertility [2]. Teratozoospermia represents a heterogeneous group that includes a broad spectrum of abnormal sperm phenotypes that affect the head, neck, midsection, and tail, alone or simultaneously [3]. The etiology of teratozoospermia has been found to be multifaceted and closely related to endocrine disorders, environmental factors, and life experiences, but little is known about the molecular defects that cause morphological abnormal spermatozoa (teratozoospermia) [4]. The identification and study of these genes are of great clinical value for the explanation of the causes of teratozoospermia, the improvement of patient management, the provision of effective therapeutic options for genetically defective patients, and the provision of adequate genetic counseling and expected therapeutic outcomes for infertile patients [5–7]. Additionally, it is essential for finding new treatments and avoiding time-consuming and painful treatments, as well as for underestimating the molecular changes in infertility. In addition, with the expanding number of clinical patients and the increasing sophistication of assisted reproductive technology (ART) [8, 9], the traditional research methods are time-consuming and inefficient, so there is an urgent need for an accurate semen diagnostic biomarker to accomplish rapid diagnosis of patients with teratozoospermia and accurately assess the success rate of assisted reproductive technologies. It suggests that we need to use more advanced technical means to carry out detailed and in-depth analysis of the molecular pathogenesis of teratozoospermia.

A number of existing studies have focused on the abnormal sperm morphology due to abnormal expression of genes related to the spermatogenesis process or loss/mutation of the Y chromosome [3, 10], including *AURKC* [11], *SPATA16* [12], *DPY19L2* [13], *DNAH1* [6], etc. Several recent studies have identified the following teratozoospermia-associated gene mutations: *FBXO43* [14], *ARMC2* [15], *SEPTIN12* [16], *AGBL5* [17], etc., by measuring exonic mutations in blood samples using whole exome sequencing technology. All of these studies have provided more in-depth analysis of the genetic basis of teratozoospermia, but their role in the clinical diagnosis of teratozoospermia is relatively weak. Several recent studies have used metabolomic and proteomic analysis to measure semen metabolites and proteins in teratozoospermia patients [18], with the expectation of identifying biomarkers that can be used for clinical diagnosis, and several differential metabolites or enzyme products have been found to be associated with teratozoospermia: antioxidative defense enzymes (AD) [19], angiotensinogen [20], nuclease activity [21], aromatase [22] and seminal ROS [23]. Some studies have shown that protein 4.1 [24], *SPATA46* [25], *CRISP2* [26], and *Spata6* [27] have the potential to become clinical diagnostic molecular markers. Although the functions of above genes or proteins are all validated in the teratozoospermia pathogenicity, they only target some specific types of teratozoospermia and are less efficient as the potential biomarkers for teratozoospermia. Therefore, there is an urgent need for an accurate semen diagnostic biomarker to complete rapid diagnosis of patients with teratozoospermia and the accurate assessment of the success rate of assisted reproductive technologies.

With the continuous development of sequencing technology and related sequencing analysis tools, high-throughput sequencing can be used to rapidly identify molecular markers for teratozoospermia, and the current research reports are limited to the screening of related differential gene clusters and the construction of related protein interaction networks [28, 29]. No study has yet reported the integration of multiple available microarray datasets using the weighted gene co-expression network analysis (WGCNA). WGCNA can be used to find clusters (modules) of highly correlated genes, to summarize such clusters using the module eigengene or an intramodular hub gene, to associate modules with other modules and external sample traits (using eigengene network methodology), and to calculate module membership measures [30]. It has been widely used to explore the large and complex relationships between microarray or RNA sequence data, which provides a convenient and effective solution for screening potential biomarkers for clinical prognosis and therapy [31, 32].

In this study, we used GSEA analysis and WGCNA analysis to effectively and deeply integrate and analyze the available microarray datasets, and finally successfully screened one differentially expressed gene. Then, we validated the expression changes of this gene using another dataset and *in vitro* experiments, which showed that the gene could be used as a diagnostic semen biomarker for teratozoospermia, which will also provide an important theoretical basis for the diagnosis and treatment of teratozoospermia.

## RESULTS

### Overview of the transcriptomes of teratozoospermia and enrichment analysis

To elucidate the molecular pathogenesis of teratozoospermia, Supplementary Figure 1A, 2B show heatmaps of all mRNAs in two datasets. From the heat map, it can be seen that the different sample groups have a good clustering effect, and the gene expression of the two groups is significantly different. Then, as shown in Supplementary Figure 1C, 2D, we constructed the volcano map using the differentially expressed mRNAs (*p*-value < 0.05). A total of 1730 mRNAs were differentially expressed in teratozoospermia in the dataset GSE6872, of which 290 mRNAs were up-regulated and 1440 mRNAs were down-regulated (|log2fold change|≥2, *p*-value < 0.05) (Supplementary Table 1); in the dataset GSE6967, there were 312 mRNAs showing differential expression in teratozoospermia, of which 118 were up-regulated and 194 were down-regulated (|log2fold change|≥2, *p*-value < 0.05) (Supplementary Table 1); in dataset GSE6968, there were 77 mRNA with expression differences in teratozoospermia, of which 60 mRNAs expressions were up-regulated and 17 mRNAs expressions were down-regulated (|log2fold change|≥2, *p*-value < 0.05) (Supplementary Table 1).

### Gene set enrichment analysis of different teratozoospermia datasets

**Figure 1:**
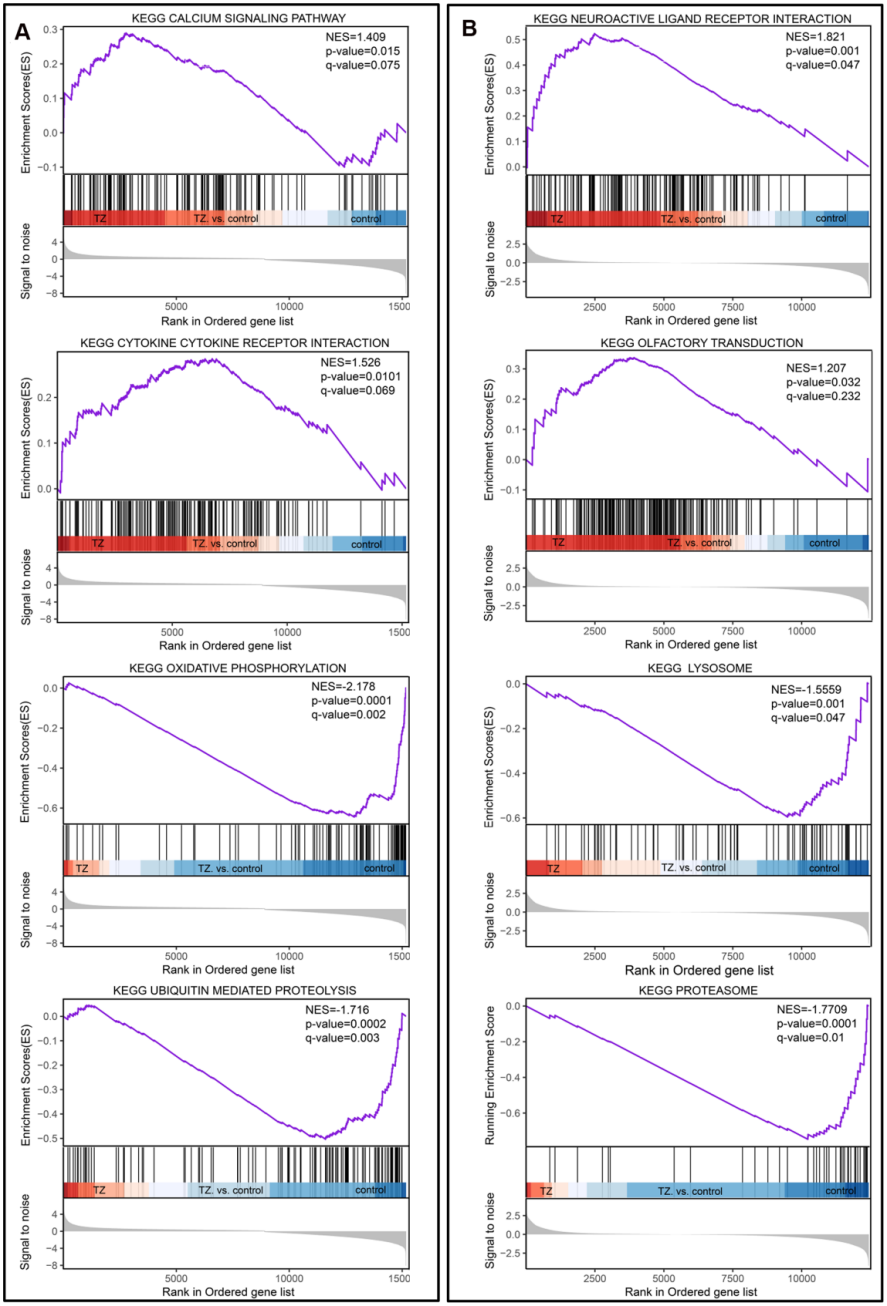
**Gene set enrichment analysis of teratozoospermia.** (**A**) The four selected pathways from the dataset GSE6872. Teratozoospermia samples were correlated positively with Calcium Signaling pathway and Cytokine Cytokine Receptor. (**B**) The four selected pathways from dataset GSE6967. Teratozoospermia samples were correlated positively with Neuroactive Ligand Receptor Interaction and Olfactory Transduction.

For the traditional analysis with DNA microarrays, the common approach involves focusing on a handful of genes at the top and bottom of L (i.e., those showing the largest difference) to discern telltale biological clues, but this approach has a few major limitations [33]. To overcome these analytical challenges, we used a method called Gene Set Enrichment Analysis (GSEA) that evaluates microarray data (GSE6872 and GSE6967) at the level of gene sets, and the results from the dataset GSE6872 showed that Calcium Signaling pathway and Cytokine Cytokine Receptor Interaction were enriched in the teratozoospermia samples; at the same time, Oxidative Phosphorylation and Ubiquitin Mediated Proteolysis were enriched in the control samples (Figure 1A). For the dataset GSE6967, Neuroactive Ligand Receptor Interaction and Olfactory Transduction were enriched in the Teratozoospermia samples; at the same time, Lysosome and Proteasome were enriched in the control samples (Figure 1B).

### Weighted gene correlation network analysis of teratozoospermia

**Figure 2:**
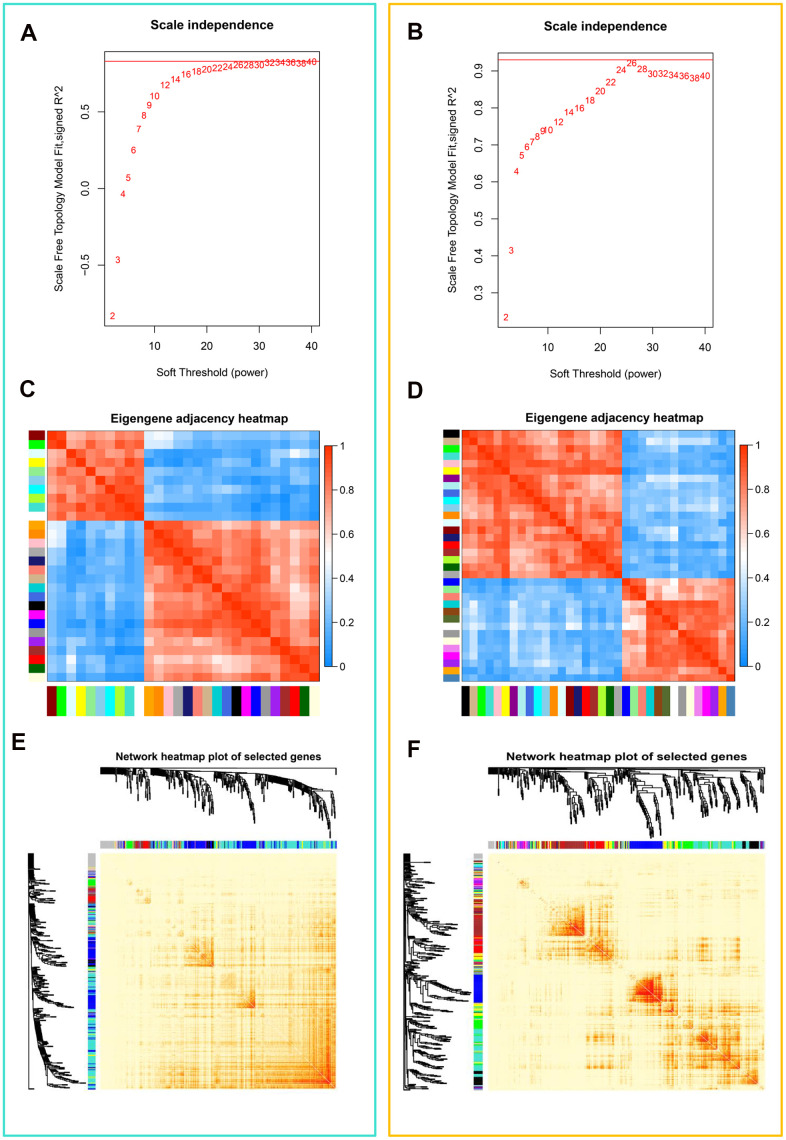
**Weighted gene co-expression network analysis (WGCNA) of genes in teratozoospermia.** (**A**) Analysis of the scale-free fit index for various soft thresholding powers (β) from the dataset GSE6872. (**B**) Analysis of the scale-free fit index for various soft thresholding powers (β) from the dataset GSE6967. (**C**) Heatmap plot of the adjacencies in the eigengene network from the dataset GSE6872. Each row and column in the heatmap corresponds to one module eigengene (labeled by color). In the heatmap, blue color represents low adjacency (negative correlation), while red represents high adjacency (positive correlation). Squares of red color along the diagonal are the meta-modules. (**D**) Heatmap plot of the adjacencies in the eigengene network from the dataset GSE6967. (**E**) Heat map plot shows the topological overlap matrix (TOM) among randomly selected 400 genes from the dataset GSE6872. Light color shows low overlap, and red color indicates higher overlap. The left side and the top side show the gene dendrogram and module assignment. (**F**) Heat map plot shows the topological overlap matrix (TOM) among randomly selected 400 genes from the dataset GSE6967.

Correlation networks are being used increasingly in bioinformatics applications. To accurately elucidate the key modules and hub genes of teratozoospermia, we used WGCNA to search for clusters (modules) and associated networks of genes that are highly associated with teratozoospermia. To ensure that scale-free networks (Figure 2A, 2B) and average connectivity remained normal (data not shown), the power of β was set to 26, which indicates that our dataset analysis had a well indexed scale-free topology. As shown in Supplementary Figure 2A, 2B, the clustering effect of these two microarray-data (GSE6872 and GSE6967) was good. Gene modules were counted, with gray modules indicating genes that could not be clustered into other modules (Supplementary Figure 2C, 2D). And modules with 29 and 35 genes were identified by the Module eigengene adjacency heatmap (Figure 2C, 2D) and hierarchical clustering dendrogram (Supplementary Figure 2E, 2F), separately from dataset GSE6872 and GSE6967. The interactions between gene modules were then analyzed and a TOM plot of the gene network was generated based on the corresponding hierarchical clustering dendrogram and modules (Figure 2E, 2F).

### Common DEGs screening and protein-protein interaction of teratozoospermia

**Figure 3:**
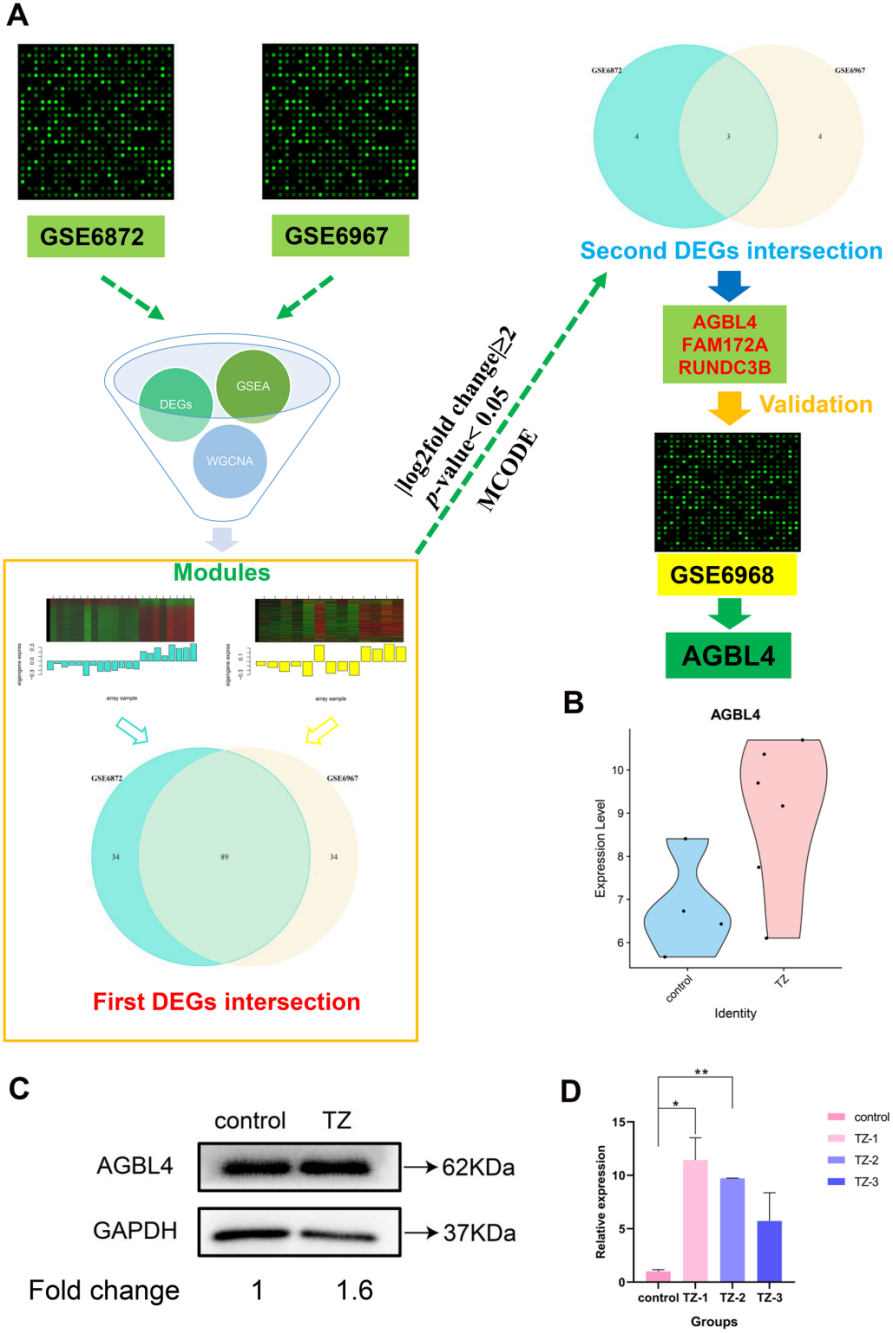
**The flowchart of the whole analysis process and *AGBL4* gene validation.** (**A**) The flowchart of the whole analysis process. After collecting the datasets GSE6872 and GSE6967, we combined differentially expressed gene screening, GSEA analysis, and WGCNA analysis to narrow down the most relevant modules (separately colored with turquoise and yellow) between the two datasets. Subsequently, after two intersections, three DEGs were screened. Then, we substituted these three differentially expressed genes into the other dataset (GSE6968) for validation and found that only AGBL4 gene had an identical expression trend to the first two datasets (GSE6872 and GSE6967). (**B**) *AGBL4* gene validation in another dataset GSE6968. TZ, teratozoospermia samples; control, healthy samples. (**C**) *AGBL4* gene expression validation using western blotting. TZ, teratozoospermia samples; control, healthy samples. The fold change calculation was finished based on gray intensities of protein bands. (**D**) *AGBL4* gene expression validation using qRT-PCR. TZ, teratozoospermia samples; control, healthy samples.

To get an insight into the function of DEGs of teratozoospermia, we made a first DEGs intersection between two datasets and screened 89 DEGs with the identical expression trend (Figure 3A and Supplementary Figure 3A, 3B) and the upregulated and downregulated DEGs were analyzed using Metascape analysis. Metascape analysis (http://metascape.org) was performed to accomplish Gene Ontology (GO) analysis to depict the unique biological significance based on DEGs between different groups [34]. After analysis, the Top 13 clusters with their representative enriched terms were shown in Supplementary Figure 1E and Supplementary Table 2, mainly including translational initiation, negative regulation of cysteine-type endopeptidase activity involved in apoptotic process, eye development, bone morphogenesis, regulation of lipid metabolic process, regulation of mitotic cell cycle phase transition, cell adhesion molecule binding, oxidative phosphorylation, primary lysosome, protein sumoylation, central nervous system neuron differentiation, regulation of neuron differentiation and positive regulation of translation. In addition, KEGG analysis showed that the common DEGs significantly were enriched in Ribosome, Proteasome, Cholesterol metabolism and Oxidative phosphorylation, etc (Supplementary Table 3). To further capture the relationships between the terms, a subset of enriched terms has been selected and rendered as a network plot (Supplementary Figure 3C), where terms with a similarity > 0.3 are connected by edges. We selected the terms with the best p-values from each of the 20 clusters, with the constraint that there are no more than 15 terms per cluster and no more than 250 terms in total. Additionally, to further investigate the common DEGs and the potential protein levels, the STRING database was applied for revealing the core PPI network (Supplementary Figure 3D).

### Identification and validation of the potential biomarkers in teratozoospermia vs. the normal controls

As WGCNA generated a large network of genes, we narrowed the network constructed from two modules (Figure 3A) to locate the hub genes common to both datasets. As a result, 89 nodes and 3916 edges were screened out for further analysis. The MCODE plugin of Cytoscape was used to screen subcluster and only one cluster was caught. Subsequently, we cross-referenced the gene set of this subcluster set with the previous total DEGs set (|log2fold change|≥2, *p*-value < 0.05) to screen for its common component. Ultimately, we screened to obtain three potential biomarker genes with the highest differential expression in the teratozoospermia group of patients, *AGBL4*, *FAM172A* and *RUNDC3B* (Figure 3A).

In order to verify the accuracy of the differential expression of the above three potential biomarker genes, we then substituted these three differentially expressed genes into the other dataset (GSE6968) for validation and found that only *AGBL4* gene had an identical expression trend to the first two datasets (GSE6872 and GSE6967), and the result (Figure 3B) proved that the *AGBL4* gene could be the diagnostic semen biomarker for teratozoospermia, which also proved the reliability of the method used in this study. In addition, the results shown in Figure 3C, 3D and Supplementary Figure 4B indicated that the expression of *AGBL4* gene was significantly higher in teratozoospermia than in normal controls, further validating the potential application of this gene in the diagnosis of teratozoospermia.

## DISCUSSION

Infertility is a major health problem that is affecting 20 million men worldwide [35]. Among male-related infertilities, teratozoospermia is one of the more common cases. For more than 30 years, sperm morphology assessment has been one of the most common tests used to assess teratozoospermia [5, 36]. However, the reliability and accuracy of the method need to be improved. Therefore, there is a requirement to develop a new method for the precise diagnosis of teratozoospermia. In this study, we integrated two datasets from the GEO database and successfully identified one significantly upregulated gene in the sperms of teratozoospermia patients, *AGBL4*. And the differential expression of this gene in another microarray dataset was further validated (Figure 3B). The above results confirmed that *AGBL4* has the potential to be a semen biomarker for teratozoospermia diagnosis.

Several studies have shown that protein 4.1 [24], *SPATA46* [25], *CRISP2* [37], *Spata6* [27] and other genes play important roles in the process of normal spermatogenesis and have potential as molecular markers for clinical diagnosis of teratozoospermia. Although the functions of the above genes have been verified, the abnormal expression of these genes is only associated with specific teratozoospermia, so they do not have a broad application prospect. In addition to the discovery of the above sperm genetic markers, some studies reported that the abnormal expression of testicular genes or the loss or mutation of the Y chromosome during spermatogenesis led to the abnormal sperm morphology, mainly including *AURKC*, *SPATA16*, *DPY19L2*, *DNAH1*, etc [10]. The above studies have provided a more in-depth analysis of the genetic basis of teratozoospermia, but do not have the potential to serve as clinical diagnostic semen molecular markers for teratozoospermia. Although some recent studies have identified multiple teratozoospermia-associated exon mutations using blood samples via whole-exome sequencing, this also provides additional possibilities for the clinical diagnosis of teratozoospermia. However, these single assays do not guarantee the accuracy of the clinical diagnosis of teratozoospermia, and the use of blood samples for the clinical diagnosis of teratozoospermia adds an additional dimension and workload compared to the molecular markers identified in this study, which makes the diagnostic process more time-consuming.

The *AGBL4* gene selected in this study encodes an ATP/GTP-binding protein [38], which is a metallocarboxypeptidase that mainly mediates the deglutamylation of target proteins, catalyzes the deglutamylation of post-translational polyglutamate side chains in proteins (e.g. tubulin), and also removes polyglutamate from the carboxyl terminus of target proteins (e.g. MYLK) [39, 40]. In addition, the protein mediates the deglutamylation of cGAS and modulates the antiviral activity of cGAS [41]. Both Cardiofaciocutaneous Syndrome 3 and Cardiofaciocutaneous Syndrome 2 have been shown to be associated with the *AGBL4* gene [42]. However, no study has yet reported that this gene is associated with male infertility, and there is only a patent indicating that *AGBL4* is one of the candidate genes for male infertility [43]. Hence, more studies are needed to further validate the role of *AGBL4* in male infertility. In this study, we further validated the differential changes of *AGBL4* gene expression in semen from different patients by means of western blotting and real-time quantitative PCR technology based on bioinformatic analysis, and the results fully demonstrated the feasibility of this gene as a clinical diagnostic marker.

In addition, several recent studies have performed a more comprehensive analysis of potential biomarkers in the seminal plasma of patients with teratospermia using metabolomic and proteomic analyses and identified several significantly differentially expressed metabolites or enzyme products. Despite the good potential of these biomarkers, metabolomic and proteomic analyses are cumbersome and expensive, which does not facilitate the widespread use of clinical diagnostics. The biomarker obtained in this study is not limited to a specific type of teratozoospermia [2], but has a broad spectrum of effect and a broad application prospect, and can be corroborated with the commonly used sperm morphological assessment, so it has important clinical significance.

In summary, this study further identifies one potential semen biomarker that could be used as clinical semen diagnostic markers for teratozoospermia using in-depth integrative analysis, and provides a theoretical basis for subsequent studies on the pathogenesis of teratozoospermia.

## MATERIALS AND METHODS

### The experimental design for the analysis

Four healthy control and 4 male patients fresh sperm samples were obtained from men with normal spermatozoa and with teratozoospermia spermatozoa at the Reproductive Medicine Center of Peking University Shenzhen Hospital. Semen was collected by masturbation after 3 days of abstinence and liquefied at room temperature for 30-60 minutes. Semen analysis was performed by a computer-assisted semen analysis system according to the World Health Organization guidelines (Supplementary Figure 4A). Study subjects were confirmed to be fathers aged 30-40 years without any treatment. The study was approved by the Ethics Committee of Peking University Shenzhen Hospital, and all participants signed a consent form allowing the use of their sperm samples in this study.

### The experimental design for the analysis

As shown in Figure 3A, after collecting the datasets, we combined differentially expressed gene screening, GSEA analysis, and WGCNA analysis to narrow down the most relevant modules between the two datasets. We then performed two intersections of DEGs to screen for potential biomarkers. Subsequently, another dataset was used to validate the expression changes of these potential biomarkers and finalize the potential biomarker.

### Data collection and preprocessing

Two datasets files were downloaded from the National Center for Biotechnology Information (NCBI) Gene Expression Omnibus (GEO) with the ID GSE6872 (including thirteen healthy controls and 8 teratozoospermia cases) and GSE6967 (consisting of five adult normal males and 8 teratozoospermia male patients). In addition, another dataset file with the ID GSE6968 (consisting of four adult normal males and 6 teratozoospermia male patients) was also prepared for subsequent validation. Robust Multichip Average algorithm (RMA) was utilized to accomplish the data normalization/standardization of these datasets. The package limma was then used to detect differentially expressed genes (DEGs) under the threshold of *p*-value < 0.05: A simple linear model was fitted to the expression matrix and empirical Bayes was used for further analysis.

### Differentially expressed genes screening

The limma package was used to screen for differentially expressed genes (DEGs) between normal and teratozoospermia spermatozoa. “|log2fold change|≥2” and “*p*-value < 0.05” was used as the judgment threshold to determine significant differences between groups. Volcano maps and heat maps were constructed using the ggplot2 package and the pheatmap package in R.

### GO enrichment analysis and Pathway enrichment analysis

Gene Ontology (GO) analysis was performed using the website (http://metascape.org/) to reveal the unique biological significance based on differentially expressed genes. Using a combination of two websites (https://david.ncifcrf.gov/ and http://kobas.cbi.pku.edu.cn/kobas3), the Kyoto Encyclopedia of Genes and Genomes (KEGG) database was used to find important pathways. The “*p*-value < 0.05” and the “|log2fold change|≥2” were used as the cutoff criterion for GO and KEGG enrichment analysis.

### Gene set enrichment analysis

Following the standard procedure for GSEA analysis [33], we first converted the expression datasets from GSE6872 and GSE6967 into tab-delimited GCT format as follows: the first column shows the gene symbols and the second column is labeled “NA”, then columns are populated with the expression values of each example. Subsequent operations were performed in full accordance with GSEA’s standard protocols (http://www.gsea-msigdb.org/gsea/).

### Protein-protein interaction network building

Differential expression of mRNA (|log2fold change|≥2, *p*-value< 0.05) were taken into the search tool to retrieve interacting genes/proteins (STRING). The confidence value was set to 0.4. The gene network files were then entered into Cytoscape software to analyze the core module of the protein-protein interaction (PPI) network using Cytoscape’s Molecular Complex Detection (MCODE) plug-in.

### Co-expression network construction

In this section, the Weighted Gene Correlation Network Analysis was performed using WGCNA package to reveal the correlation between genes. First, DEGs (*p*-value < 0.05) was input into R software to detect good genes and samples. To ensure that the network is scale-free, the power of the β is set to 26. The minimum number of modules is 11. Hierarchical clustering dendrogram summarized the gene modules of different colors. Heat maps and topology overlap matrix (TOM) diagrams were used to visualize the module structure.

### Common DEGs Selection between two datasets and Validation in another dataset

First, the gene network files exported from the WGCNA analysis were input into the Cytoscape software. The K-core values for each subcluster were then calculated using the MCODE plugin of Cytoscape. After that, we intersected the DEGs in the most correlated modules from the two datasets (GSE6872 and GSE6967) and selected the common DEGs. We then intersected the common DEGs using the cutoff criterion (“*p*-value < 0.05” and the “|log2fold change|≥2”) from the two datasets (GSE6872 and GSE6967) to screen for potential biomarkers. Subsequently, another dataset GSE6968 was used to validate the expression changes of these potential biomarkers and finalize the potential biomarker.

### Biomarker gene validation experiments *in vitro*

After screening for the potential biomarker gene, semen samples from teratozoospermia and normal controls were collected for further validation. Expression detection of the potential biomarker gene was performed by western blotting and qRT-PCR according to the previous protocol [44, 45]. For western blotting, the primary antibodies used were: AGBL4 (1:500, Affinity Biosciences, OH, USA, Cat# DF3981) and GAPDH (1:2,000, Affinity Biosciences, Cincinnati, OH, USA, Cat# AF7021). And the secondary antibody used was: horseradish peroxidase (HRP)-conjugated goat anti-rabbit (1:1,000, Beyotime, Catalog no. A0208). Chemiluminescence was then performed using a BeyoECL Star Chemiluminescence Kit (Beyotime, Catalog no. P0018AS) and photographs were taken under enhanced chemiluminescence (ECL) detection system (ProteinSimple, San Jose, CA, USA). As for the qRT-PCR, total RNA from purified spermatozoa was extracted with TRIzol Reagent (Invitrogen) and the primer sequences were as follows: GAPDH, forward primer 5’ ACAACTTTGGTATCGTGGAAGG -3’, reverse primer 5’-GCCATCACGCCACAGTTTC-3’; AGBL4, forward primer 5’-ATGAGGAACGGTTCCAGAGGCA-3’, reverse primer 5’- GCAATAGGAAGTGTGGTCCAGG-3’.

### Data availability statement

The authors confirm that the data supporting the findings of this study are available within the article and its supplementary materials.

## Supplementary Material

Supplementary Figures

Supplementary Table 1

Supplementary Table 2

Supplementary Table 3
