# Supplementary material for: One potential biomarker for teratozoospermia identified by in-depth integrative analysis of multiple microarray data
Source: Aging (Albany NY). 2021 Mar 26;13(7):10208–24. doi: 10.18632/aging.202781 (PMC8064145; doi:10.18632/aging.202781)
Supplement: Supplementary Figures [file aging-13-202781-s001.pdf]

SUPPLEMENTARY FIGURES

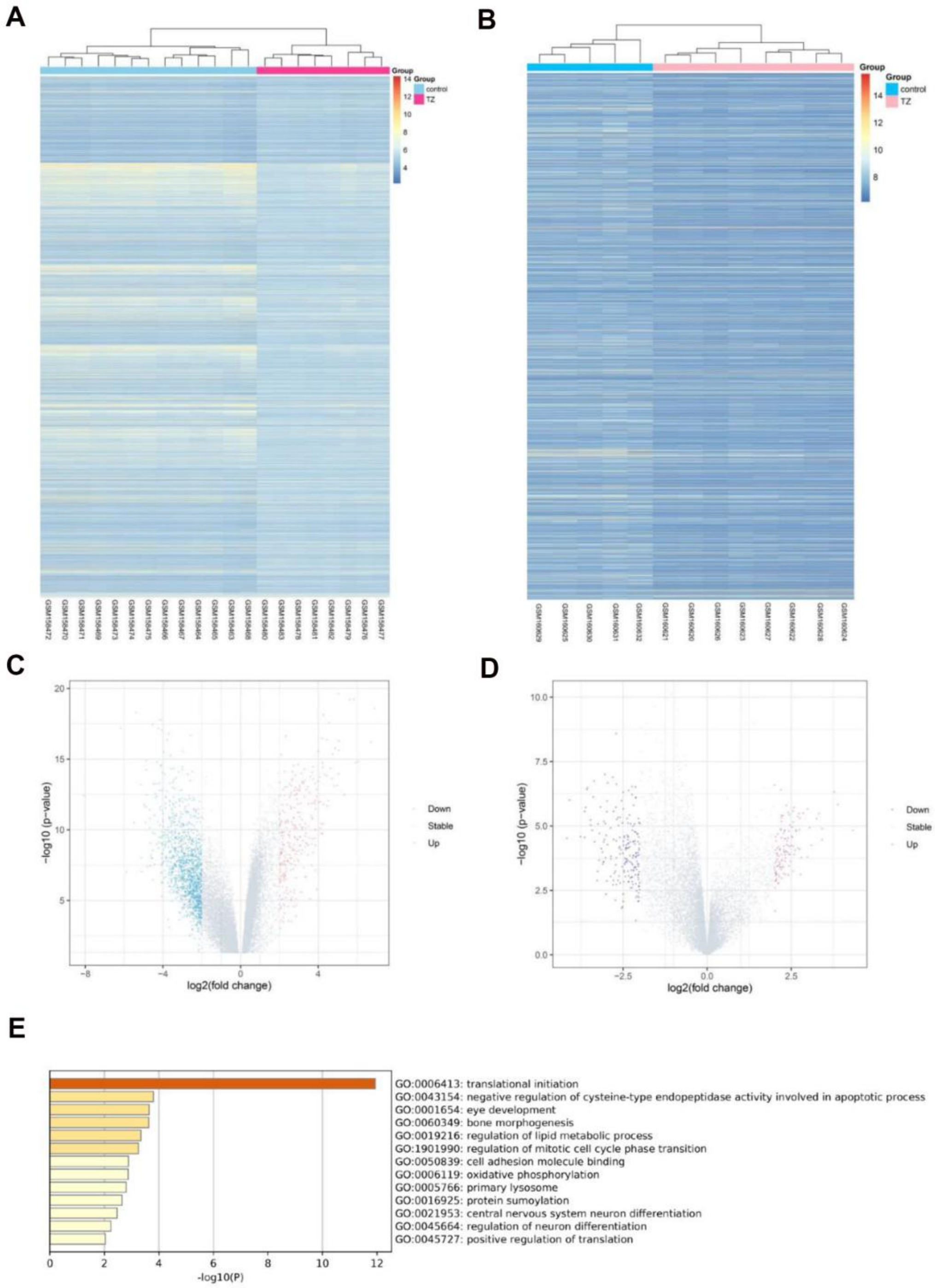

Supplementary Figure 1. Overview of the Transcriptomes of Teratozoospermia and enrichment analysis of common 89 DEGs. (**A**) Heat map of all mRNAs detected by RNA-Seq from the dataset GSE6872. (**B**) Heat map of all mRNAs detected by RNA-Seq from the dataset GSE6967. (**C**) Differentially expressed genes (*p*-value < 0.05) were plotted in volcano with different colors, including upregulated (red) and downregulated mRNAs (blue) from the dataset GSE6872. (**D**) Differentially expressed genes (*p*-value < 0.05) were plotted in volcano with different colors, including upregulated (red) and downregulated mRNAs (purple) from the dataset GSE6967. (**E**) Top 13 clusters with their representative enriched term based on GO enrichment analysis of differentially expressed genes (DEGs).

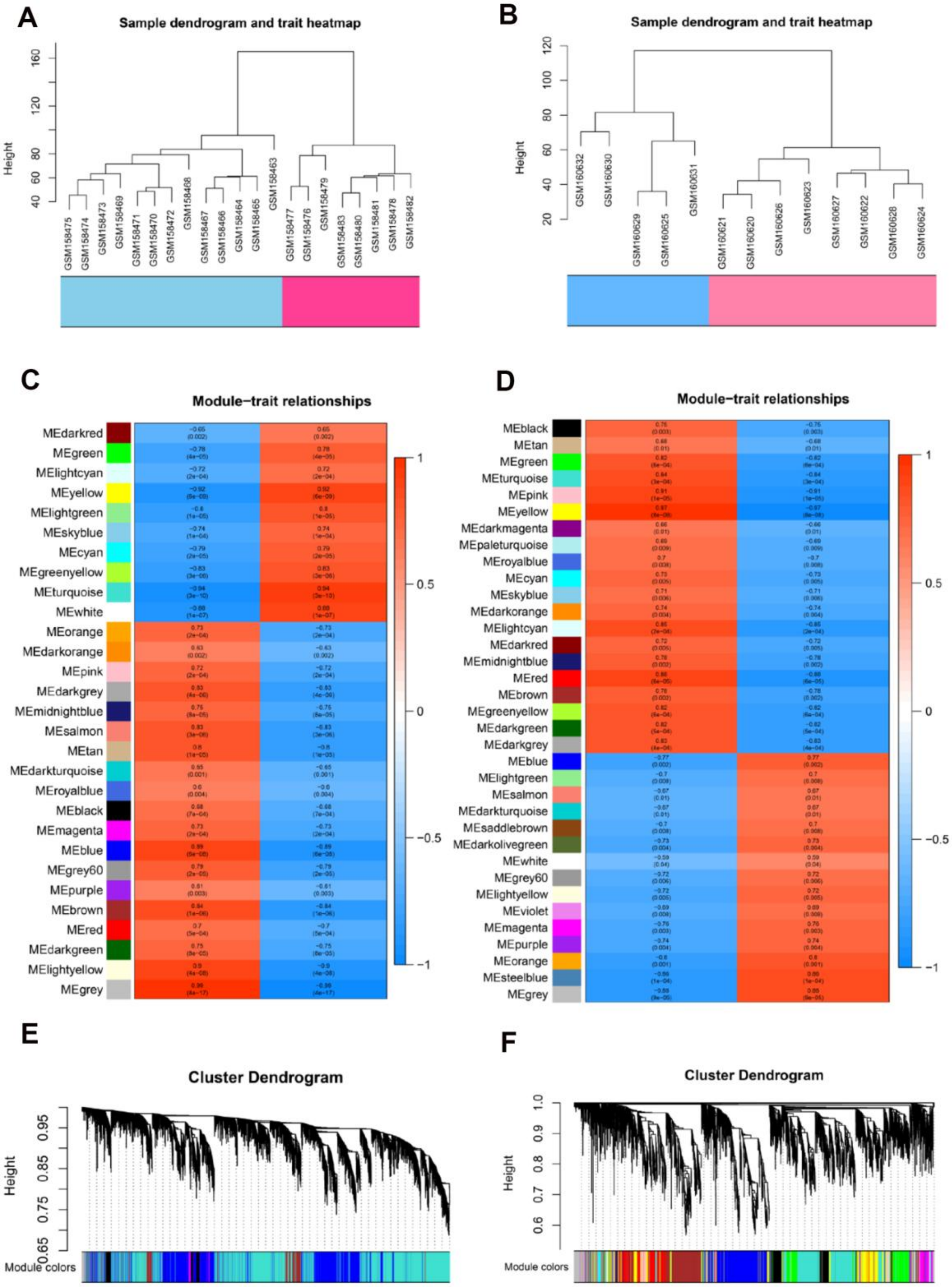

Supplementary Figure 2. Weighted gene co-expression network analysis (WGCNA) of genes in teratozoospermia. (**A**) Cluster dendrogram displays the relationship between different samples from the dataset GSE6872. (**B**) Cluster dendrogram displays the relationship between different samples from the dataset GSE6967. (**C**) The module trait relationships based on WGCNA analysis of genes in teratozoospermia from the dataset GSE6872. Gene modules were calculated, and the gray module represents genes that cannot be clustered into any other modules. (**D**) The module trait relationships based on WGCNA analysis of genes in teratozoospermia from the dataset GSE6967. Gene modules were calculated, and the gray module represents genes that cannot be clustered into any other modules. (**E**) Hierarchical cluster tree showing co-expression modules identified by WGCNA from the dataset GSE6872. (**F**) Hierarchical cluster tree showing co-expression modules identified by WGCNA from the dataset GSE6967.

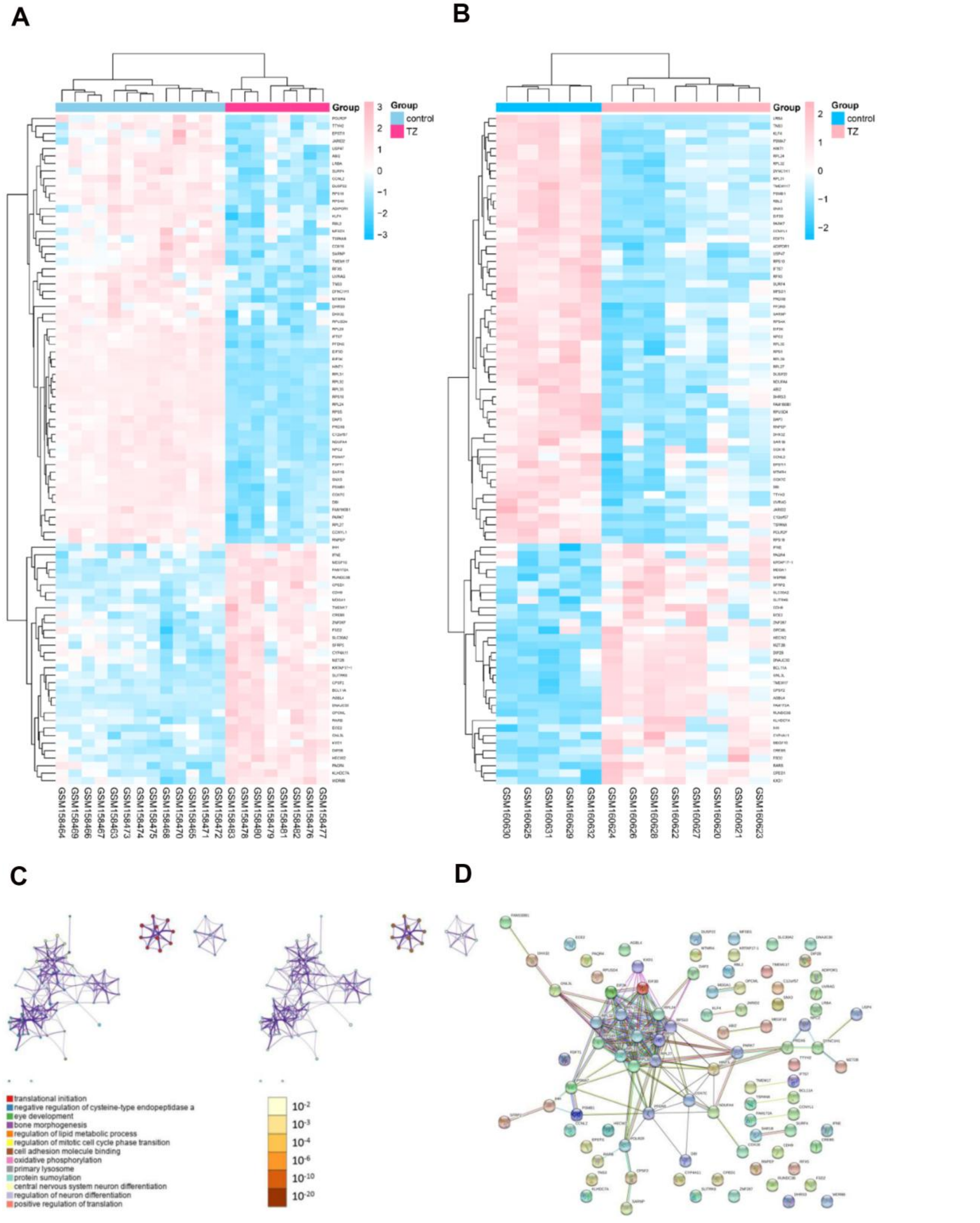

Supplementary Figure 3. The heat map, enrichment analysis and Protein-protein interaction (PPI) networks based on these 89 common mRNAs. (**A**) Heat map of 89 common mRNAs detected by RNA-Seq from the dataset GSE6872. (**B**) Heat map of 89 common mRNAs detected by RNA-Seq from the dataset GSE6967. (**C**) Network plot of relationships between subsets of selected enriched terms. The terms were selected with the best p-values from each of the 20 clusters, with the constraint that there are no more than 15 terms per cluster and no more than 250 terms in total. (**D**) The PPI network was constructed by STRING based on 89 common differentially expressed mRNAs (*p*-value< 0.05) that were input into STRING, the confidence score was set at 0.4.

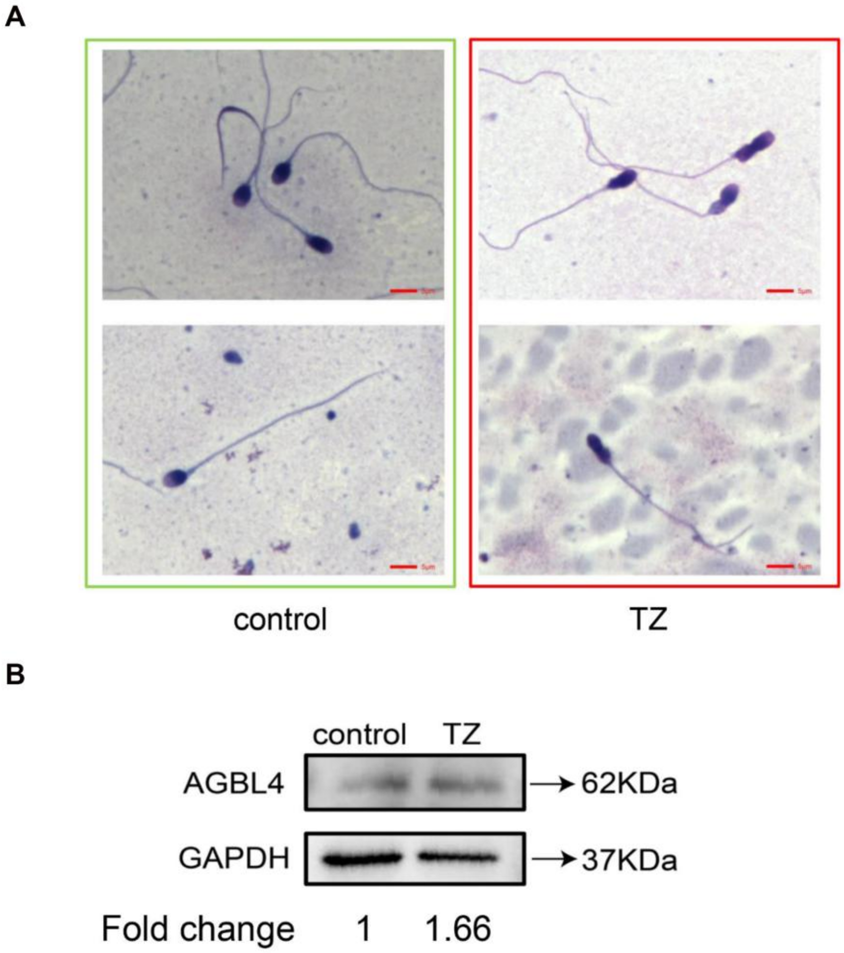

Supplementary Figure 4. The Semen analysis of different samples and the *AGBL4* gene expression validation using western blotting. (**A**) Morphology of collected sperm under microscopy. (**B**) *AGBL4* gene expression validation using western blotting. TZ, teratozoospermia samples; control, healthy samples. The fold change calculation was finished based on gray intensities of protein bands.
